# Developing a Safer Conception Intervention for Men Living with HIV in South Africa

**DOI:** 10.1007/s10461-017-1719-4

**Published:** 2017-02-13

**Authors:** Hazar Khidir, Christina Psaros, Letitia Greener, Kasey O’Neil, Mxolisi Mathenjwa, F. N. Mosery, Lizzie Moore, Abigail Harrison, David R. Bangsberg, Jennifer A. Smit, Steven A. Safren, Lynn T. Matthews

**Affiliations:** 1 Harvard Medical School, Boston, MA USA; 2 Behavioral Medicine, Department of Psychiatry, Massachusetts General Hospital, Boston, MA USA; 3 Maternal Adolescent and Child Health (MatCH) Research Unit, Department of Obstetrics and Gynaecology, University of the Witwatersrand, Durban, KwaZulu-Natal South Africa; 4 Center for Global Health, Massachusetts General Hospital, 125 Nashua Street, Suite 722, Boston, MA 02114 USA; 5 London School of Hygiene and Tropical Medicine, London, UK; 6 Department of Behavioral and Social Sciences, Brown University School of Public Health, Providence, RI USA; 7 Division of Infectious Diseases, Massachusetts General Hospital, Boston, MA USA; 8 Discipline of Pharmaceutical Sciences, College of Health Sciences, University of KwaZulu-Natal, Durban, South Africa; 9 Department of Psychology, University of Miami, Miami, FL USA

**Keywords:** Safer conception, HIV prevention, Reproductive health, Behavior change, Intervention development, MLWH, HIV-serodiscordant couples, South Africa, Qualitative research

## Abstract

Within sexual partnerships, men make many decisions about sexual behavior, reproductive goals, and HIV prevention. There are increasing calls to involve men in reproductive health and HIV prevention. This paper describes the process of creating and evaluating the acceptability of a safer conception intervention for men living with HIV who want to have children with partners at risk for acquiring HIV in KwaZulu-Natal, South Africa. Based on formative work conducted with men and women living with HIV, their partners, and providers, we developed an intervention based on principles of cognitive-behavioral therapy to support men in the adoption of HIV risk-reduction behaviors such as HIV-serostatus disclosure and uptake of and adherence to antiretroviral therapy. Structured group discussions were used to explore intervention acceptability and feasibility. Our work demonstrates that men are eager for reproductive health services, but face unique barriers to accessing them.

## Resumen

Entre parejas sexuales, el hombre toma muchas decisiones sobre el comportamiento sexual, las metas reproductivas y la prevención del VIH. Hoy a pesar de llamados para involucrar a los hombres en la salud reproductiva y la prevención del VIH. Este artículo describe la creación y evaluación de la aceptabilidad de una intervención de concepción más segura para hombres viviendo con VIH que desean tener hijos con parejas seronegativas en KwaZulu-Natal, Sudáfrica. Basándonos en el trabajo formativo realizado con hombres y mujeres viviendo con VIH, sus parejas y proveedores, desarrollamos una intervención basada en los principios de la terapia cognitiva-conductual para apoyar a los hombres en la adopción de comportamientos de reducción del riesgo de VIH, como la revelacion de su estatus, aceptacion y adherencia a la terapia antirretroviral. Se utilizaron conversaciones estructuradas en grupo para explorar la aceptabilidad y factibilidad de la intervención. Nuestro trabajo demuestra que los hombres están ansiosos por los servicios de salud reproductiva, pero enfrentan barreras para acceder a ellos.

## Introduction

Many people living with HIV (PLWH) want to have children [1]. South Africa has the largest population of PLWH in the world, and the majority are of reproductive age [2]. Moreover, HIV-serodiscordance amongst stable South African couples is highly prevalent [3]. Given that over 60% of new HIV infections in sub-Saharan Africa are estimated to occur in stable, serodiscordant sexual partnerships [4] and serodiscordant couples risk HIV transmission to achieve pregnancy [5, 6], intended conception likely represents a significant contributor to incident HIV infections [7]. However, HIV prevention counseling for PLWH and their partners rarely addresses their reproductive desires [8, 9].

Advances in HIV prevention, improvements in access to HIV care resulting in longer life expectancies, and growing recognition of reproductive rights for PLWH make provision of comprehensive reproductive health services a growing priority [10, 11]. Though guidelines outlining safer conception strategies like antiretroviral therapy (ART) for the infected partner, pre-exposure prophylaxis (PrEP) for the uninfected partner, condomless sex timed to ovulation, and semen processing technologies exist [11–13]; health care workers lack basic training and system-level support on how to counsel clients, so most PLWH never receive safer conception counseling [8, 9, 14–16]. Men are even less likely to be offered reproductive counseling [17].

Many decisions around contraception and HIV prevention are determined by the male partner [1, 18]. Men informed about reproductive health are more likely to support partners in decisions on contraception and employ HIV risk reduction strategies [18]. Thus, providing reproductive health interventions to both men and women is critical to ensuring that MLWH protect their health as well as that of their families. Recognition of the need to include men in reproductive health services has grown; however, whether MLWH are interested in accessing reproductive health counseling and how to approach and structure male-inclusive interventions remains uncertain [19].

Prior work has shown that MLWH are motivated to have healthy (i.e. HIV-negative) children, are interested in counseling that helps them safely meet reproductive goals, and modify risk behavior once pregnancy is achieved to protect the baby from HIV acquisition [20–24]. This led us to propose a safer conception intervention that focused on men living with HIV in order to reduce and/or modify male-driven HIV transmission risk behavior. We developed a male-focused cognitive behavioral therapy (CBT)-based intervention to encourage and support MLWH who want to have children with HIV-exposed partners to adopt safer conception behaviors, including HIV-serostatus disclosure and initiation of ART. Prior reproductive health interventions have promoted contraception [25–27], adherence to PMTCT [28–31], and increased antenatal HIV testing [17, 32–35] by working with African men and couples. Only one other published study has described developing a safer conception intervention delivered to South African couples [36]; however, none have aimed to explicitly work with men to implement safer conception strategies.

Given the complexities and challenges of this work, we describe the systematic, iterative design and content of a novel pilot safer conception intervention for men based on formative research studies and feedback from potential participants regarding feasibility and acceptability. We then describe the next steps for ongoing research to evaluate the intervention. We hope that this data will inform the efforts of other researchers and implementers working to engage men in HIV prevention and reproductive health efforts.

## Sequential Methods and Results

### Formative Research

We conceptualized the design of our intervention based on a series of formative research studies conducted between 2010 and 2015 (Table 1)with either HIV-positive men and women reporting a recent pregnancy with HIV-negative or serostatus-unknown partners or providers at public-sector clinics in eThekwini District, South Africa where antenatal clinic HIV prevalence is estimated at 41% [37].

**Table 1:** Important findings from formative studies with key stakeholders

Studies with stakeholders	Methods	Key findings
Qualitative studies with men and women living with HIV [20, 21, 40]	In-depth interviews explored periconception HIV risk knowledge and practices and experiences accessing reproductive counseling	• Men and women living with HIV want to have children and this is often prioritized over HIV prevention • Pregnancies were rarely explicitly planned • Men drive many decisions regarding condom use and conception plans • Had incomplete understanding of HIV-serodiscordance • Had limited knowledge on how to reduce sexual HIV transmission during condomless sex • Barriers to accessing counseling: difficulty initiating conversations around reproductive goals during clinic visit and fear of judgment from health care providers • Expressed interest in receiving counseling to reduce periconception transmission risks • Described modifying HIV risk behavior once pregnancy was achieved in order to protect the baby
Quantitative study with men and women living with HIV [7, 22]	Cross-sectional survey on prevalence of periconception risk behavior	• Initially attempted to recruit recent pregnancy partners from HIV-infected women at an antenatal clinic; however, no women were able to bring their male partners • A large percentage of women (41%) and men (32%) screened for participation did not know their recent pregnancy partner’s HIV serostatus • 4% of women and 13% of men were in a mutually-disclosed serodiscordant relationship • 40% of HIV infected women and 27% of HIV infected men had disclosed their serosatus to a recent pregnancy partner • ART and condom use were reported by participants; however, none engaged in these strategies as safer conception methods • None reported timing condomless sex to peak fertility or endorsed using semen washing or manual insemination
Studies with health care workers [38, 39]	In-depth interviews and focus group discussions explore provider practices of assessing fertility intentions and providing safer conception advice	• Most providers did not routinely assess reproductive goals among women or men living with HIV • Providers expressed incomplete knowledge of serodiscordane and HIV risk reduction strategies other than condoms • Most providers had never offered patients safer conception counseling, citing discomfort toward offering harm reduction counseling • Nurses and doctors reported time constraints as additional challenges to offering more comprehensive safer conception counseling
Periconception HIV-risk behavior conceptual framework [65]	Adapted the Information-Motivation-Behavioral Skill Model using formative research and the literature to identify individual, structural, and couple-level determinants of safer conception behavior	• High unemployment, cultural gender ideologies around manhood, and HIV-related stigma mean that men wield greater decision-making power within relationships • Implementing safer conception interventions requires an individual to understand HIV transmission and risk reduction, have skills to effectively communicate with their partner, and have sufficient motivation and behavioral skills to implement safer conception strategies • Dyadic factors such as couple communication dynamics significantly impact pregnancy intentions and partner involvement in safer conception strategies

We found that HIV prevention counseling in public sector health facilities centers on condom use and strategies to prevent mother to child transmission (PMTCT), offering little-to-no counseling on how to minimize periconception transmission risk [21, 38–40]. Nurses, counselors, and physicians in public sector clinics rarely provide safer conception counseling due to low knowledge about serodiscordance and safer conception strategies, discomfort with promoting risk reduction behaviors that condone condomless sex, and time constraints [38]. With limited access to reproductive counseling, we found that HIV-positive individuals with recent pregnancy often misunderstand HIV- serodiscordance, do not disclose their status to their pregnancy partner, and are unfamiliar with periconception HIV risk reduction [7, 22].

### Developing the Safer Conception for Men Intervention

**Fig. 1:**
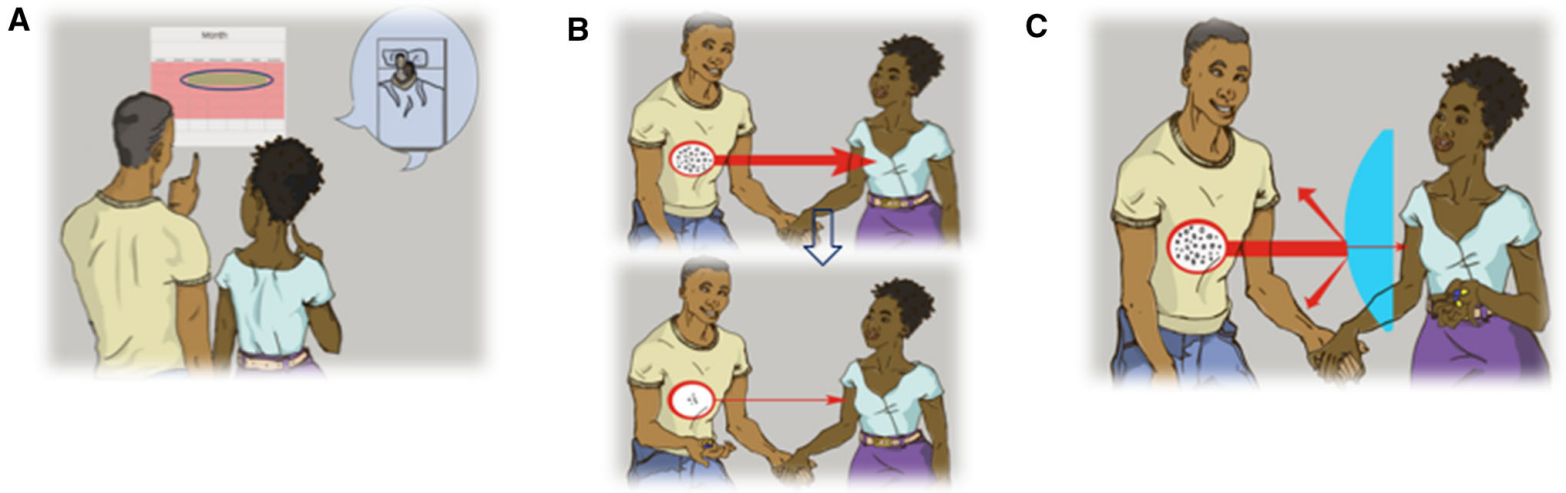
Locally relevant images used to present key safer conception strategies including **a** Timed condomless sex to peak fertility, **b** Treatment as Prevention, **c** Pre-exposure Prophylaxis for the HIV-uninfected apartner

Based on our findings that MLWH have poor knowledge of serodiscordance and safer conception behaviors, we provided comprehensive safer conception education in our intervention. We drafted didactic information that defined HIV-serodiscordance, addressed common myths about serodiscordance, and explained HIV transmission risks associated with serodiscordance. We also provided education on the range of safer conception strategies recommended by international and national guidelines, including: (a) Couples HIV Counseling and Testing (CHCT), (b) initiation of ART and delaying condomless sex until achieving viral load suppression (treatment as prevention), (c) timing condomless sex to peak fertility, (d) daily oral TDF-based PrEP, (e) treatment of STIs, (f) sperm washing and in vitro fertilization, and (g) adoption. To aid in conveying this information, we created a counseling tool integrating locally-relevant illustrations to explain key concepts (Fig. 1).

Because lowering HIV transmission risk involves behavior change we determined that an effective safer conception intervention would impart behavioral skills, including communication skills to facilitate disclosure, overcome barriers to behavior change, and address motivation for behavior change.

**Fig. 2:**
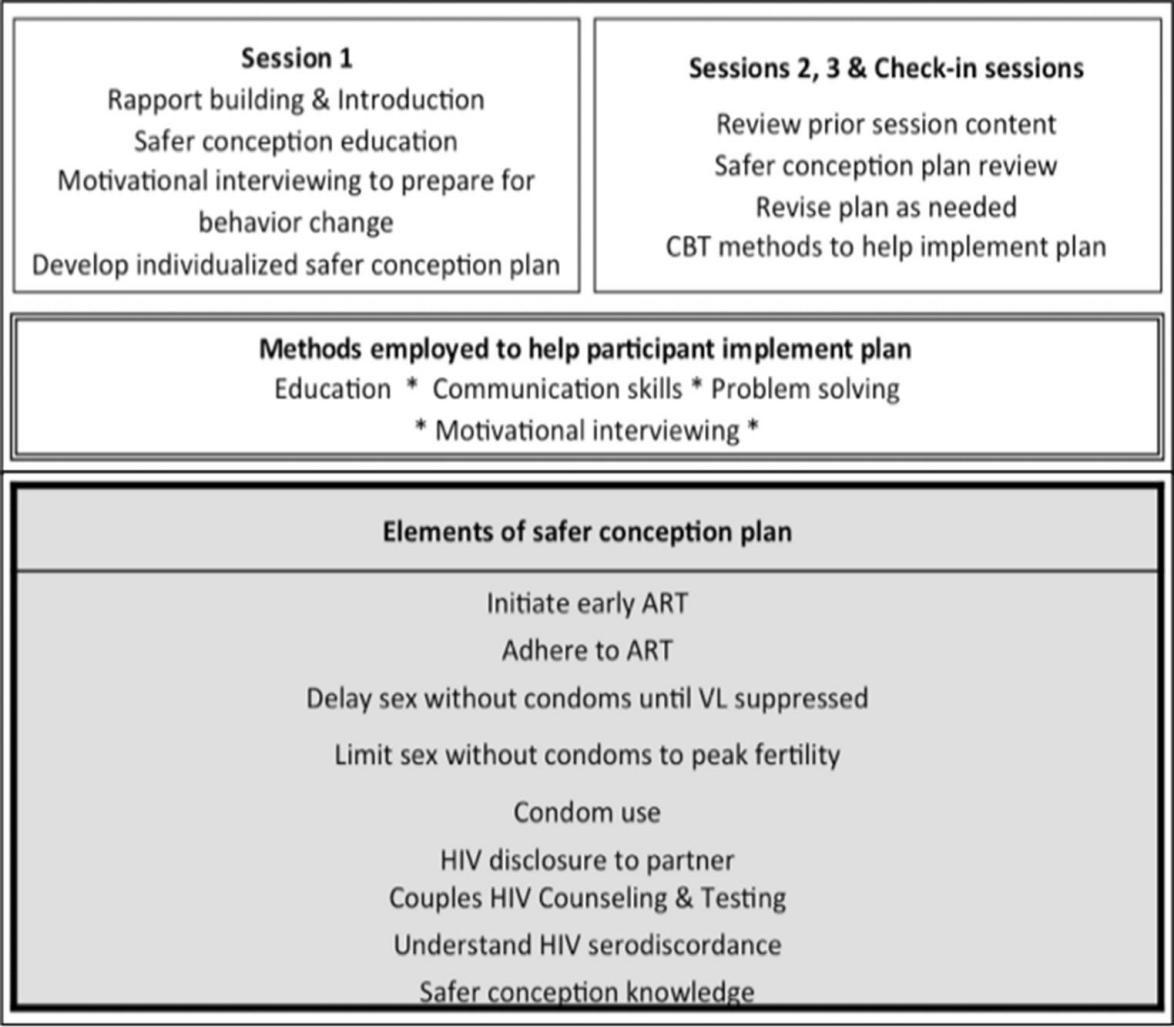
Safer conception intervention structure and content

Integration of cognitive behavioral therapy (CBT) techniques into the intervention was led by clinical and research psychologists (CP, SAS) with experience designing, implementing, and testing behavioral modification techniques for ARV adherence and HIV prevention [41–43]. Motivational interviewing and problem solving skills were adapted from the Life-Steps intervention [42]. Communication skills training was based on elements from the Stepping Stones [44–46] and Horizons program both developed in South Africa and aimed to foster forthright and respectful communication between partners and support participants to negotiate disclosure and fertility goals [47, 48]. We combined educational and CBT content into a multi-session intervention consisting of three core sessions and two follow-up sessions (Fig. 2).

In Session 1, participants were introduced to the five counseling sessions, participated in safer conception education, discussed motivation to change behavior related to HIV transmission in order to conceive, and began to generate a safer conception plan. Educational content focused on HIV-serodiscordance and risk reduction, explored the risks and benefits of having children based on each man’s circumstances, and included contraceptive options given the high incidence of unintended pregnancy [22, 49].

A motivational interviewing exercise was used in Session 1 to prepare participants for behavioral change, and incorporated into subsequent sessions when needed (e.g., when challenges to behavior change were encountered). Once participants articulated safer conception strategies they wished to pursue (referred to as their Healthy Baby Plan), subsequent sessions (Sessions 2–5) were individually tailored to be relevant to helping the participant achieve his goals. Problem-solving skills were incorporated into sessions to address anticipated and actual barriers to implementing safer conception strategies, including adherence to ARVs (for individuals for whom that was part of the safer conception plan). Lastly, communication skills training was incorporated into the intervention to help participants approach negotiations on disclosure, fertility goals, and healthy baby plan cooperation with their partners. As disclosure of HIV-serostatus to pregnancy partner was deemed critical and required for successful deployment of many safer conception strategies, all participants received counseling on the importance of disclosure and training on specific disclosure skills. Disclosure counseling included a discussion of the pros and cons of disclosure, brainstorming and problem-solving common barriers to disclosure, and communication skills exercises.

Follow-up sessions were designed to provide support to participants as they executed their safer conception plan. The goal of these sessions was to assess participants’ progress in carrying out their Healthy Baby Plan and identify and solve problems that arose over the course of the intervention sessions (i.e. end or change in partnership, breakdowns in partner communication, change in fertility goals or Healthy Baby Plan).

### Implementing a Multi-phase Study to Evaluate Acceptability, Feasibility, and Efficacy of Our Intervention

**Fig. 3:**
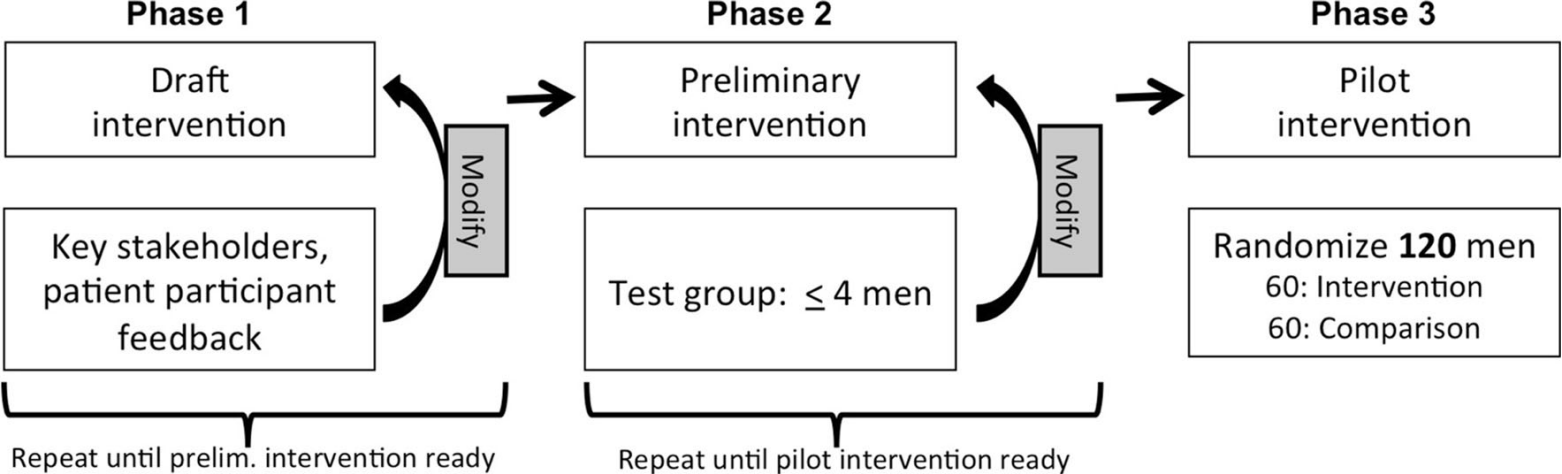
Iterative intervention development study design schema

To evaluate our preliminary intervention, we designed a multi-phase iterative pilot study (Fig. 3). In the first phase of the study, we conducted structured group discussions with HIV-infected men who wanted to have a child with an uninfected or unknown-serostatus partner in the next year in order to refine intervention content and evaluate acceptability of the pilot intervention. We are currently conducting an open pilot to trial our intervention and further refine intervention content, enhance acceptability, and further gauge feasibility (Phase II). In the final phase of our iterative process, we plan a randomized control pilot. In our randomized pilot, we aim to compare our intervention against a time-matched control standard of care condition, comparing likelihood of HIV-RNA suppression at 6 months across the two groups. We will also evaluate uptake of and adherence to ART, serostatus disclosure to partner, communication, and gender norms.

### Ethics

The competing concerns regarding respecting the privacy of the male participant and doing our best to protect female partners were carefully considered and will be outlined in a subsequent publication. We created letters for men to give to their pregnancy partners informing them of their risk for acquiring HIV and encouraging them to seek HIV counseling and testing. Men were reminded that these letters would effectively disclose their HIV-serostatus to the partners. We offered men the opportunity to bring their female partners to counseling sessions to encourage transfer of safer conception counseling to partners though we recognized that not all men, particularly those that had yet to disclose, would be willing to do this. We created a separate information session for those female partners who were able to participate. Ethics approvals for Phase 1 and Phase 2 of the study were obtained from the Human Research Ethics Committee at the University of Witwatersrand (Johannesburg, South Africa) and the Institutional Review Board at Partners Healthcare (Boston, USA). Site permission was also obtained from the study clinic.

## Target User Evaluation of Preliminary Intervention

### Methods

A structured group discussion format was selected to facilitate input from a greater number of individuals than could be achieved with individual interviews and to explore community norms. We recruited men aged 20–45, who had known their HIV-serostatus for at least 6 months, were receiving HIV care but not yet on ART, reported a stable HIV-negative or unknown-serostatus partner, and reported interest in having a child with this partner in the next year. Recruitment was carried out at an NGO/DOH collaborative healthcare facility in a large township immediately outside Durban, South Africa. Group discussions were led by a female research associate with extensive experience in qualitative data collection and supported by a male co-facilitator [21, 38, 50, 51]. Discussions were conducted in isiZulu and audio-recorded; audio-recordings were transcribed and translated to English. The transcripts were independently coded by three researchers and qualitatively analyzed through thematic analysis using an iteratively-developed codebook to explore emergent themes. Key data points were summarized then discussed by the team and compared for consistency and discrepancies.

The perceived effectiveness, feasibility, and acceptability of intervention content was explored in our group discussions. Findings on MLWH’s perceptions of individual safer conception strategies will be described elsewhere. Here we report on the overall acceptability of our intervention and whether MLWH would attend the sessions, the most appropriate setting for our intervention, and perceived barriers to intervention uptake.

## Results

**Fig. 4:**
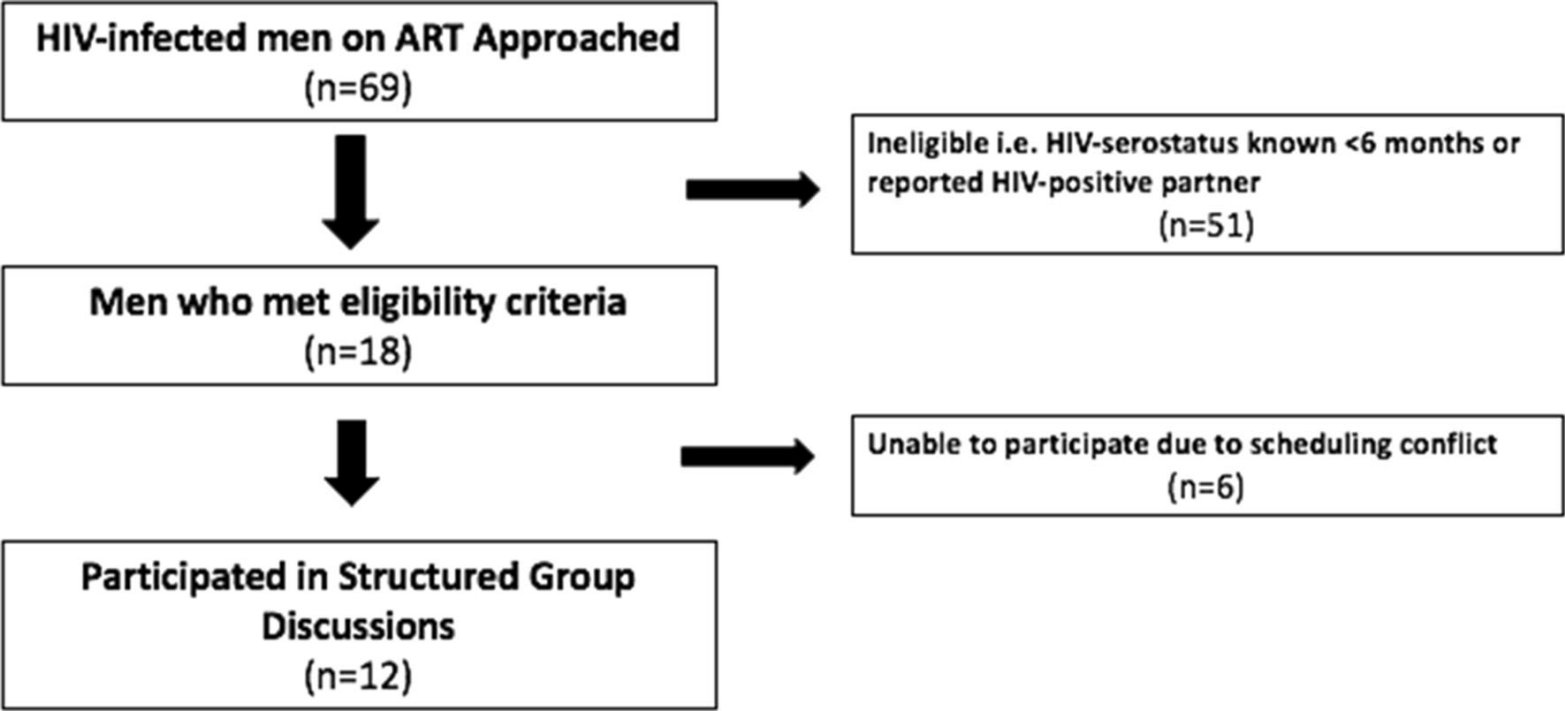
Structured group discussions recruitment flowchart

We experienced challenges in recruiting and retaining men for our structured group discussions. Many men were ineligible because they had known their HIV serostatus for fewer than 6 months or reported an HIV-infected partner; moreover, six of the men who met eligibility criteria could not attend the group discussions due to work scheduling conflicts (Fig. 4). Although group discussions did not include the number of participants that constitute a typical focus group discussion, we conducted structured group discussions with the participants who were available, employing a focus group discussion format.

A total of 12 clients participated in three structured group discussions that were comprised of 3–5 men. Enrolled participants were median 37-years-old, 50% had completed secondary school, 75% were employed, and all identified as black South Africans. Participants reported a median of 2 current sexual partners (range 1–4), 1 desired pregnancy partner (range 1–3), nearly half reported that they did not know their intended pregnancy partner’s HIV-serostatus, 33% had disclosed their serostatus to an intended pregnancy partner, and 85% endorsed using condoms at their last sexual encounter with their pregnancy partner(s).

## Willingness to Access a Safer Conception Intervention at a Healthcare Facility

Across the three group discussions (GD), participants described interest in receiving information on strategies they could use to conceive and excitement at the idea of a safer conception resource.

P3: “That [how to have children without transmitting HIV to partners] is exactly what we want to know. As to what can be the protection for the baby from being infected, as well as how the female can be protected.” –GD1

When moderators queried participants on whether they would attend a multi-session counseling intervention that would support them in protecting a partner from HIV to thus produce an HIV-uninfected child, consensus across groups was that MLWH would attend multiple sessions because they would be eager to gain information and advice on safer conception strategies. Most participants felt that the clinic was the most appropriate setting for participating in safer conception counseling because they valued the privacy afforded by a clinic setting and perceived safer conception services as important to their overall health.

P10: “I recommend the clinic because when I am here, it is where I get hope for my situation. … I think the clinic is the right place because this is also as important as my life.” –GD3

## Perceived Barriers to Uptake

Participants described a number of challenges to participating in the proposed intervention. Several participants noted that many men living with HIV could benefit from the intervention but would not take up the resource because they are apprehensive to engage in HIV care.

P2: “…there are many men nowadays who want to [have children] but they do not speak out, that is a challenge for men. Because, my sister, this is very helpful there are many men who wish for this. I really do not know how we can do that so that this gets to have a wide range because there are so many who want to have babies but are afraid to speak out.” –GD1

P3: “Even [at the clinic], people who you find mostly are women. There are fewer men who come here, you do not find them because they are scared.” –GD1

Participants also reported that men don’t have enough information about HIV and what care is available and called for increasing community awareness about safer conception strategies. Participants cited difficulties scheduling sessions around employment responsibilities as a practical challenge to the proposed multi-session intervention. This was reflected in the challenges we encountered in scheduling focus group discussions around the men’s varied work schedules.

## Acceptability of Engaging Female Partners

Participants who had disclosed their status to their partner reported that it would be feasible for men to bring their partners to counseling sessions, citing that it would provide opportunities to explain their intentions, to initiate new reproductive strategies, and recruit her to participate in safer conception plans. However, participants who had not yet disclosed felt that it would be very challenging to engage partners.

P10: “I would be able to bring her because our life is based on a condom. So if we have to stop using a condom without having explained to her or if she finds out from me she might not trust this strategy. She might think I want to put her in trouble, but if she comes with me and get the same information and counseling and when we do what we will be doing in whatever method I think she might also feel comfortable and perhaps that baby could be a success because we will both be focused on one thing.” –GD3

P4: “It is very difficult because she does not know that I am already infected.” –GD2

### Integrating Findings into the Intervention

Qualitative data from structured group discussions were reviewed and the findings used to modify the intervention. The aim was to finalize several features of the intervention including: session format, intervention setting, and session content. Given the challenge of scheduling group discussions with multiple men and the challenges each unique man’s situation presented (e.g. disclosure, partner serostatus, partnership type, communication skills, attitudes towards ART), sessions were formatted as individual (rather than group) sessions. This format also allows for provision of counseling specifically tailored to each participant’s individualized pregnancy and HIV prevention plan. A clinic setting was determined to be an appropriate environment to carry out intervention sessions based on group feedback and practical considerations.

Initially, we anticipated the possibility of honing educational content to decrease session length. However, findings from our groups revealed a general lack of knowledge about important biological HIV concepts (i.e. viral load, CD4 count), family planning techniques, and HIV prevention strategies (Matthews 2016, submitted). Structured group discussion participants also expressed eagerness for more information on safer conception methods. Based on this feedback, we added basic information about HIV, CD4 cell count, ART, and HIV-RNA. We opted to offer participants the option of including female partners in the sessions based on overall consensus from participants that this would be an acceptable element of the intervention.

Once intervention content was finalized, we developed an intervention manual to formalize and structure intervention content and guide interaction between interventionist and participant. We chose to train HIV counselors to deliver intervention content as clients are accustomed to receiving other HIV counseling services from lay counselors. The counselors, who were lay counselors with the equivalent of a high school education, were trained over five days on general counseling skills, the use of CBT strategies in effecting health-related behavior change, and the intervention content. Counselors then participated in supervision calls every two weeks with a study investigator (CP), during which time cases were reviewed in detail and feedback on the intervention was provided. A local clinical psychologist was brought onsite to further support counselors on counselling and CBT skills. Site visits were also conducted for continued training/supervision and problem-solving issues related to intervention delivery.

## Discussion

Development of the Men’s Safer Conception Intervention was a 5-year, iterative process that revealed key insights into the reproductive health demands of MLWH. Important insights gained from our mixed-methods studies with HIV-infected individuals in confirmed- and potentially-serodiscordant relationships include that PLWH risk sexual HIV transmission to meet fertility goals, men often dominate decisions around use of risk-reduction strategies, and that healthcare providers rarely share safer conception opportunities with men. These findings revealed the need for programs that engage men in reproductive health. Our findings that MLWH expressed eagerness for more reproductive health programming and modify their HIV-risk behavior to protect their offspring suggest that men are willing to participate in a safer conception intervention and provided an opportunity to motivate men to modify HIV risk behaviors. We developed an educational CBT intervention consisting of three core sessions and two follow up sessions. The intervention provides comprehensive education on safer conception strategies, encourages men to adopt an explicit plan to implement safer conception strategies, and uses behavioral skills training to support men as they begin to implement their Healthy Baby Plan. After developing our intervention content, we used a multiphase participatory research approach to refine our intervention [52]. The intervention was well-received by structured group discussion participants from our target population. MLWH expressed excitement toward the prospect of a safer conception resource and willingness to attend clinic over multiple sessions for the purpose of accessing reproductive health resources. This finding questions historical assumptions that this population is not interested in seeking these resources [18, 53, 54].

Despite largely positive FGD feedback toward our intervention, implementation of our safer conception intervention is likely to face a number of challenges. MLWH may find it logistically difficult to schedule multiple sessions as noted by our group discussion participants. More importantly, as was noted in our group discussions, though MLWH may desire safer conception counseling, many men are not engaged in HIV care. Our formative studies recruited men who were already engaged in HIV care; however, our intervention will likely most benefit many men who are not receiving any HIV care. Men are less likely to access ART and achieve suppressed HIV RNA, are more likely to be lost to follow-up, and have higher rates of mortality related to HIV than women [55–57]. Novel approaches are needed to bring men in contact with HIV care. Offering men services to have healthy babies is a novel approach to drawing men to access HIV care and treatment services. Supporting men to meet important personal and sociocultural reproductive goals may increase demand for services and thus support increases in HIV testing, engagement in care, uptake of ART, and HIV RNA suppression. Providing safer conception services to men may also promote disclosure and link HIV-exposed, at-risk women to HIV prevention opportunities. Additionally, to better reach men who do not wish to seek services in a clinic setting, we plan to recruit men using mobile clinics in our subsequent intervention study phases. We hope to refine and hone our recruitment strategy to work with those not yet in care in future iterations of our intervention.

Our structured group discussion participants endorsed willingness to adopt safer conception strategies and indicated that they would be willing to invite their female partners to participate in the counseling intervention. However, it remains unclear to what extent men will deploy these behaviors. This will be examined in our ongoing pilot of the intervention. Piloting our intervention will provide a more rigorous assessment of the effectiveness of a male-focused behavioral intervention in modifying HIV-risk behavior and reducing periconception transmission risk. Piloting our intervention will also allow us to assess the feasibility of our use of lay counselors to deliver our intervention content. CBT interventions for HIV care have traditionally been delivered by providers with more training, such as nurses [41, 58, 59].

Our iterative intervention development process is subject to a number of limitations. Because of the qualitative nature of our study, some of our formative studies, including our informal group discussions, had small sample sizes that may not be representative of the larger South African population or generalizable to other populations. Participants in our formative studies may have had a particular interest in this topic that is not shared by the greater population. Acceptability and feasibility findings should also be interpreted with consideration of participant social desirability bias. Finally, study participants in our formative studies and group discussions were men who were already engaged in HIV services and sought care at public-sector clinics; thus, their views may not represent those of men who are not linked to HIV services or clinical care.

In summary, our experience developing and evaluating a safer conception intervention represents the first attempt to develop a safer conception intervention for MLWH. As the reproductive health community urges greater engagement from men [53, 60–64], it is essential to both implement reproductive health interventions to actively engage men and share strategies for developing effective interventions for this important population. Though recent policy recommendations call for interventions that engage men in sexual and reproductive health services, it is likely that lingering biases, wariness, and assumptions toward the role of men in reproductive health have resulted in sluggish echoes of this call in the HIV medical and academic community. Challenging the status quo regarding reproductive health intervention delivery by developing new approaches to increase male engagement in reproductive health issues will be important to supporting MLWH to protect their own health and the health of their families.
